# Comparison of plugin and redundant marker sets to analyze gait kinematics between different populations

**DOI:** 10.1186/s12938-023-01177-w

**Published:** 2023-12-12

**Authors:** Run Ji, Wayne Yuk-wai Lee, Xinyu Guan, Bin Yan, Lei Yang, Jiemeng Yang, Ling Wang, Chunjing Tao, Shengzheng Kuai, Yubo Fan

**Affiliations:** 1 School of Biological Science and Medical Engineering, School of Engineering Medicine, Beijing Advanced Innovation Centre for Biomedical Engineering, Beihang University, Beijing, 100191 China; 2 Department of Orthopaedics and Traumatology, The Chinese University of Hong Kong, Hong Kong SAR, China; 3 Key Laboratory of Human Motion Analysis and Rehabilitation Technology of the Ministry of Civil Affairs, National Research Center for Rehabilitation Technical Aids, Beijing, 100176 China; 4 Department of Spine Surgery, Shenzhen Second People’s Hospital, Shenzhen, 518039 China; 5 Department of Spine Surgery, First Affiliated Hospital of Shenzhen University, Shenzhen, 518035 China; 6 Shenzhen University School of Medicine, Shenzhen, 518060 China; 7 Shenzhen Youth Spine Health Center, Shenzhen, China; 8 Tianjin Key Laboratory for Advanced Mechatronic System Design and Intelligent Control, School of Mechanical Engineering, Tianjin University of Technology, Tianjin, 300384 China; 9 National Demonstration Center for Experimental Mechanical and Electrical Engineering Education, Tianjin University of Technology, Tianjin, China

**Keywords:** Inverse kinematic algorithm, Kinematics, Marker set, Gait model

## Abstract

**Background:**

Gait model consists of a marker set and a segment pose estimation algorithm. Plugin marker set and inverse kinematic algorithm (IK.) are prevalent in gait analysis, especially musculoskeletal motion analysis. Adding extra markers for the plugin marker set could increase the robustness to marker misplacement, motion artifacts, and even markers occlusion. However, how the different marker sets affect the gait analysis's kinematic output is unclear. Therefore, this study aims to investigate the effect of marker sets on the kinematic output during level walking in different populations.

**Results:**

In all three planes, there are significant differences (*P* < 0.05) between marker sets in some kinematic variables at the hip, knee, and ankle. In different populations, the kinematic variables that show significant differences varied. When comparing the kinematic differences between populations using the two marker sets separately, the range of motion (ROM) of hip flexion was only found to be a significant difference using the redundant marker set, while the peak internal rotation at the knee was only found a significant difference using plugin marker set. In addition, the redundant marker set shows less intra-subject variation than the plugin marker set.

**Conclusion:**

The findings in this study demonstrate the importance of marker set selection since it could change the result when comparing the kinematic differences between populations. Therefore, it is essential to increase the caution in explaining the result when using different marker sets. It is crucial to use the same marker set, and the redundant marker set might be a better choice for gait analysis.

## Background

Gait analysis is the systematic measurement and assessment to characterize human locomotion. Kinematic and kinetic data could be acquired through gait analysis to provide quantitative information. Clinicians could apply gait analysis to assess patients' motor dysfunctions. However, lots of research [1–5] usually found contradictory results when conducting gait analysis. Gait model selection might be one of the main reasons.

The conventional gait model (CGM), a generic name for a group of similar gait models, emerged in the 1980s [6–8] and is widely used in clinical and clinical research. The CGM was designed with the least markers, allowing quick preparation and computation as fewer markers are needed to track. The segments' position and orientation were determined by captured markers directly. However, the CGM has some limitations. Specifically, the captured data might be invalid if any one or more markers were obscured during walking or other human movements. In addition, the misplacement of the thigh markers could lead to an erroneous definition of the coronal plane of the femur, affecting the calculation of joint kinematics [9]. Moreover, the length of segments was not fixed and varied by as much as 2 cm during walking, which prevented the use of CGM in more advanced modeling techniques such as muscle length modeling that required rigid linked segments [10].

To overcome the CGM limitations, an optimized model of a cluster of markers on each segment of the lower extremities (6DOF) emerged [11]. Each cluster has at least three noncolinear markers attached to each segmental skin. Anatomical markers of the hip, knee, and ankle were firstly applied to define the segments' pose during the static trial. Then these anatomical markers were recreated based on the specific marker clusters during dynamic trials [11, 12]. The segments' pose was determined by these virtual anatomical markers during walking or other human movements. Since 6DOF tracks markers are placed on the rigid clusters, only three markers are essential to determine the rigid clusters' pose, although these clusters usually consist of more than three markers in practical. Therefore, the data are also valid, although one or two markers of one cluster are obscured during walking because the missing markers could also be recreated based on the remaining at least three markers located on the cluster. Otherwise, the rigid clusters could reduce soft tissue artifact as there should be no motion of the tracking markers relative to each other for one cluster. Thus, 6DOF might be more accurate, robust, and repeatable when compared to the CGM [11–13]. However, 6DOFs still did not impose constraints on segment length.

Modern inverse kinematic techniques (IK) [14–16], a kind of kinematic fitting or global optimization, may be a potential approach to incorporate advanced modeling techniques since this technique assumes constant segmental length during human movements. First, this approach defines a linked segment rigid body model. An optimization method (often referred to as the weighted least square optimization) was applied to minimize the sum of the squared differences between the experimental markers and their corresponding markers on the defined rigid body model. The joint kinematics calculated through IK were more robust and less sensitive to noise when compared to CGM and 6DOF [14]. The IK tends to be more popular since it was incorporated with advanced modeling techniques such as OpenSim [17] and AnyBody [18].

Previous studies [13, 14, 19–25] have tried to investigate the agreement of joint kinematics between these gait models. Slight differences in sagittal plane angles of the lower extremities and slightly larger differences in the hip and knee transverse plane angles were found between two different marker sets of CGM [19]. Similar findings have been reported for the Comparison of the CGM and 6DOF. The main differences between the two models occurred in the hip rotations, transverse knee angles, and coronal knee angles [12]. In addition, the mean differences above 5 degrees and maximum differences greater than 10 degrees were reported in hip rotation, knee rotation, and knee abduction/adduction [24]. Differences in joint kinematics between the CGM and IK could be up to 13 degrees [22].

Collectively, the kinematic output for different gait models indeed shows inconsistent results. The result from IK is the closest to the actual values [14]. Hence, IK is becoming increasingly popular for musculoskeletal research and gaining interest in the clinical community. Apart from the segment pose estimation algorithm, the marker set is also an essential element of the gait model. The Plugin marker set is the most commonly used for IK because it requires least markers. Nevertheless, more markers for marker set have the strength of insensitive marker misplacement, soft tissue artifact, and marker obscuring, especially when the segmental pose estimation applies IK since IK is a global optimization of the distance between experimental markers and markers on the musculoskeletal model. However, whether more markers for the gait model affect the kinematic output is not clear when implementing IK. Further, whether the different marker sets affect the kinematics in different populations during activities of daily living such as level walking?

Therefore, this study aims to investigate the effects of the common-used plugin marker set and redundant marker set on gait kinematics in different populations using IK. Two hypotheses were proposed, (1) some of the gait kinematics may be affected significantly when using redundant marker set, and (2) some of the kinematic differences between populations may be affected significantly when using different marker sets.

## Methods

### Subjects

Power analysis of previous study’s data indicated that 16 participants were needed to achieve 80% statistical power with alpha level of 0.05 [effect size = 1.37, an error = 0.05, power = 0.80] via G*power software (version 3.1.9.2) [26]. Nine healthy subjects (age = 38.67 ± 3.00 years,height = 158.00 ± 5.13 cm,weight = 60.22 ± 4.29 kg,BMI = 23.73 ± 0.65 kg/m^2^) and nine OA patients (age = 64.67 ± 5.81 years, height = 155.50 ± 6.05 cm,weight = 61.89 ± 6.23 kg,BMI = 25.13 ± 1.78 kg/m^2^) were recruited to participate in this study (Table 1). The inclusion criteria for healthy subjects are as follows: (a) no visible motor dysfunction, (b) no history of lower extremities injuries, (c) no any complaints of pain. Those nine subjects were assigned to Healthy Group. In contrast, the inclusion criteria for OA patients are as follows: (a) diagnosed with OA in the medial tibiofemoral compartment in right side or bilateral side and the Kellgren & Lawrence (KL) score greater than two based on radiography, (b) ability to walk without other aids, (c) with the complaints of pain, (d) no cardiovascular or neurological disease, (e) no lower limb surgery within the last year. Those nine patients were assigned to OA Group. In this study, six out of the nine patients were diagnosed with KL 3, whereas the remaining three were classified as KL 4. Among the patients, four had bilateral OA, while the remaining five had unilateral OA. The KOOS pain score were 21.13 ± 7.27. This study was approved by Shenzhen Second People's Hospital in China (Approval Number: 033Q-01PJ). All participants gave their informed consent before trial.

**Table 1:** Demographic data for all subjects

	Healthy subjects	OA patients	*P*-value
Age (year)	38.67 (3.00)	64.67 (5.81)	< 0.01**
Height (cm)	158.00 (5.13)	155.50 (6.05)	0.39
Weight (kg)	60.22 (4.29)	61.89 (6.23)	0.35
BMI (kg/m)	23.73 (0.65)	25.13 (1.78)	0.06
KL = 3/4	–	6/3	–
OA Bilateral side/Right side	–	4/5	–
KOOS pain	–	21.13 (7.27)	–

**p-value < 0.1, KOOS = Knee Osteoarthritis and Outcome Score, KL = Kellgren-Lawrence grade

### Marker sets and procedures

**Fig. 1:**
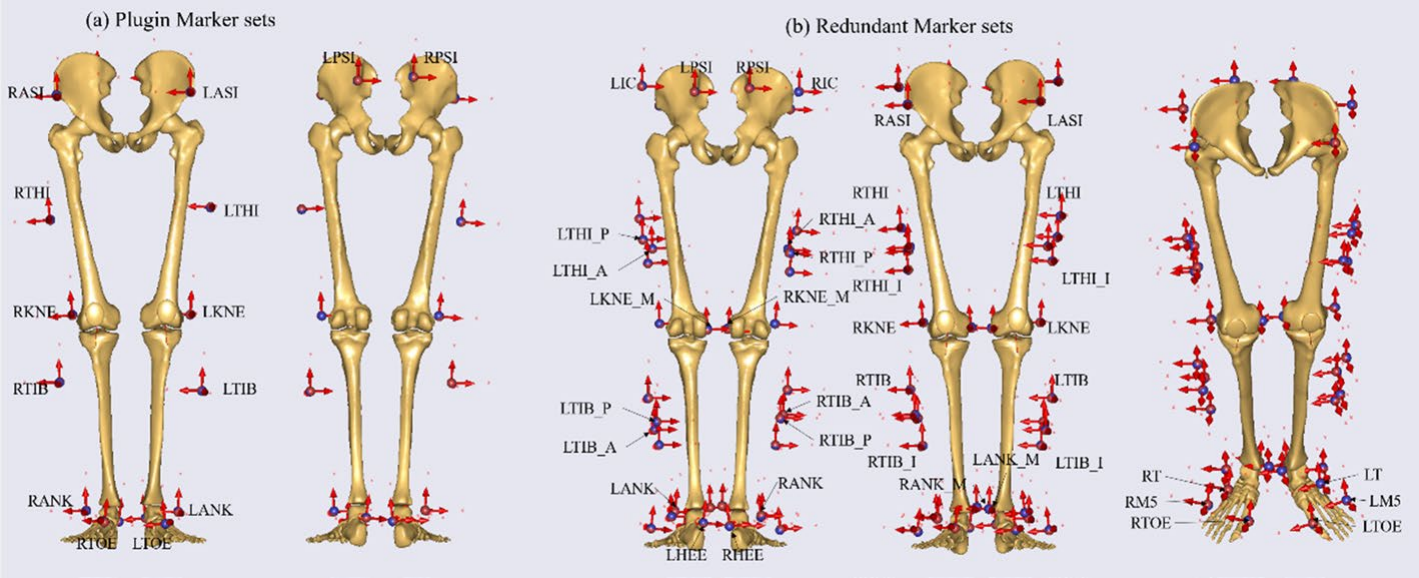
**a** Marker locations for Plugin marker set; **b** Marker locations for Redundant marker set

Plugin marker set (including 16 markers) was a commonly used marker set. Based on previous study [27], 22 extra markers were selected and applied to compose the redundant marker set in this study (Fig. 1). In the left side, the 11 additional markers were placed on the inferior, anterior and posterior to the marker placed on the left thigh (LTHI) in the Plugin marker set (LTHI_I, LTHI_A, LTHI_P), inferior, anterior and posterior to the marker placed on the left tibial (LTIB) in the Plugin marker set (LTIB_I, LTIB_A, LTIB_P), left iliac crest (LIC), medial epicondyle of the femur (LKNE_M), dorsal aspect of the left 5th metatarsal (LM5) and dorsal aspect of the left tarsal (LT). In the right side, the extra markers' placement is similar to the left side.

Before the experiment, 38 Retro-reflective markers were placed on the participants' skin surfaces according to the definition of the redundant marker set. They walked barefoot at a self-selected, roughly constant speed for several minutes for practicing. Then data collection started. The participants kept standing in the anatomical pose for several seconds to get a standing reference trial. Afterward, they repeated walking for data collection until three good trails, defined as contact with each force platform with only one foot. Gait data were measured using a 10-camera motion analysis system (MotionAnalysis Corp., CA, USA) and two force platforms (AMTI Inc., Watertown, MA, USA) at 100 Hz.

### AnyBody musculoskeletal model and simulation

A lower extremities musculoskeletal model was created for each participant using the AnyBody modeling system (AMS, version 6.0.6, Aalborg, Denmark) to estimate joint angles. Specifically, joint angles were computed by minimizing the least-square differences between markers on the musculoskeletal model and experimental markers on the participants' body [18]. In this study, the musculoskeletal models had a total of 22 degrees of freedom (DOF), including 2 × 2 DOF at the ankle joints, 2 × 3 DOF at the knee joints, and 2 × 3 DOF at the hip joints, and 6 DOF at the pelvis.

In default of AMS, the Plugin marker set was applied to match and drive the musculoskeletal model. In this study, Redundant marker set and Plugin marker set were used to match and drive the musculoskeletal model separately.

The musculoskeletal model was scaled using a standing reference trial for each participant. The pelvic width, thigh length, shank length, foot length, and initial joint angles were calculated using the least-square minimization method based on the standing reference trial. The estimated segmental length and joint angles were applied to get the offset between every marker on the musculoskeletal model and its subordinate segment. The redundant marker set contained 38 markers' offset, while the plugin marker set contained 16 markers' offset. The offset of the same marker name between the two marker sets was consistent. Therefore, the lower extremities musculoskeletal model and 38 markers' offset make up the Redundant-Gait-Model (R-model) (Fig. 1b). The lower extremities musculoskeletal model and 16 markers' offset make up the Plugin-Gait-Model (P-model) (Fig. 1a).

In one trail, the 38 experimental markers were matched with the 38 markers on the musculoskeletal model to calculate the joint angles for the R-Model. In the same trail, the 22 extra markers were removed from the experimental data and the musculoskeletal model. The remaining 16 experimental markers were matched with the remaining 16 markers on the musculoskeletal model to calculate the joint angles for the P-Model.

### Data analysis

**Fig. 2:**
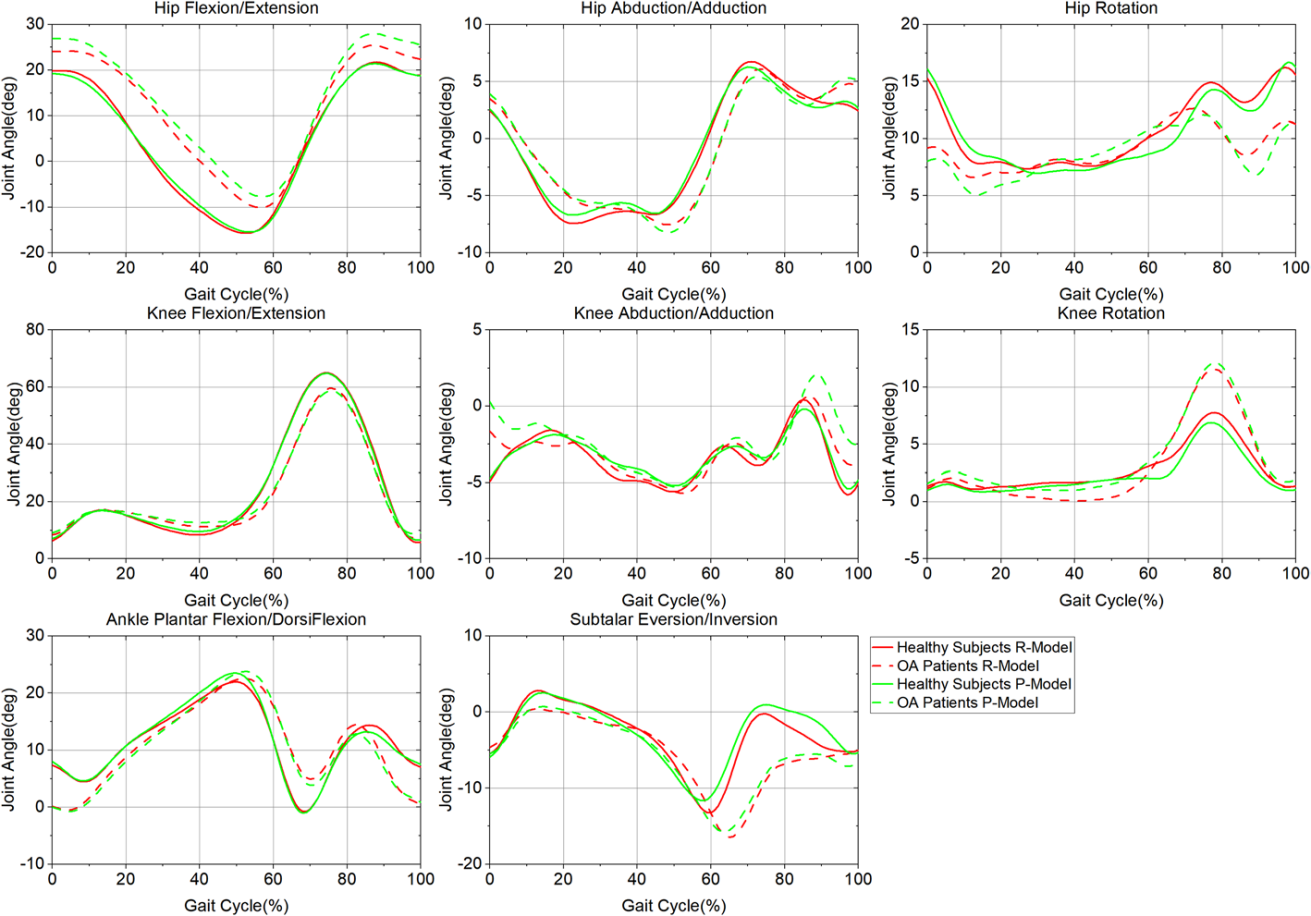
Right leg joint angle curves throughout the gait cycle, mean of the nine healthy subjects or the nine OA patients. Red dot line = averaged joint angle of nine healthy subjects using Redundant-Gait-Model, Red dashed line = averaged joint angle of nine OA patients using Redundant-Gait-Model, Green dot line = averaged joint angle of nine healthy subjects using Plugin-Gait-Model, Green dashed line = averaged joint angle of nine OA patients using Plugin-Gait-Model

In this study, all the joint angles were time-normalized to one gait cycle and resampled using spline interpolation by 0–100% with 101 points. The gait cycle was defined as the time interval between the adjacent heel strikes of the right leg. For visual comparison, joint angle curves were plotted (Fig. 2). The mean absolute variability (MAV) [28], i.e., maximum minus minimum values along each normalized point averaged over the gait cycle, was calculated for each variable. The kinematics involved maximum joint angle, minimum joint angle, ROM of joint angle throughout the gait cycle, joint angle when heel strike, and joint angle of every time point during gait.

Normality was tested with Shapiro–Wilk’s tests. Paired *t*-tests were conducted to detect MAV and the kinematic differences between two marker sets. Independent *t*-tests were applied to detect the demographics and kinematics between groups. The significance level for all analyses was set at *P* < 0.05. Data analysis was performed using a custom-made program implemented in MATLAB (The MathWorks, Inc.).

## Results

### Agreement between models in two groups – joint angle during the gait cycle

**Fig. 3:**
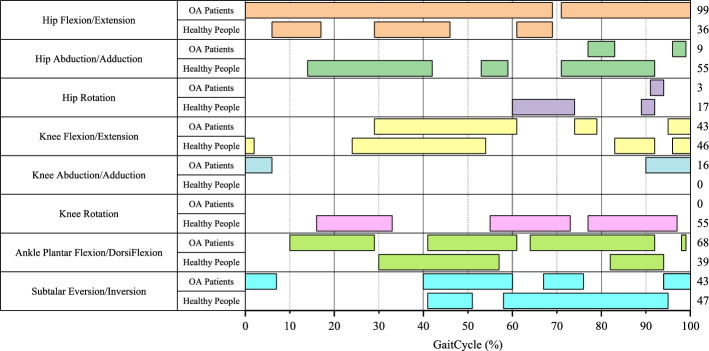
Significant difference of joint angle between models for every point throughout gait cycle

Figure 2 shows that the two groups demonstrated different joint angle curves between models throughout the gait cycle. In the same group, the joint angles are also different between models at some points (Fig. 3). Hip Flexion/Extension showed a significant difference (*P* < 0.05) between models for 99 points in OA patients while only 36 points in healthy subjects. All the joint angles except Knee Rotation were found to have significant differences (*P* < 0.05) ranging from 3 to 99 points between models in OA patients. In comparison, all the joint angles except Knee Abduction/Adduction were found to have significant differences (*P* < 0.05) ranging from 17 to 55 points between models in OA patients.

### Agreement between models and between groups–kinematic variables

**Fig. 4:**
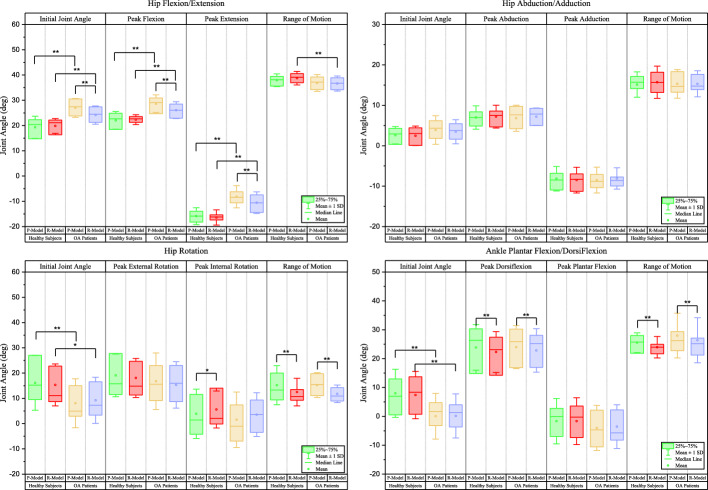
Box plot of the comparison of kinematic variables of the hip and Ankle joints across different marker sets and populations. Initial joint angle: joint angle when heel strike of the right leg. Range of motion: range of joint angle throughout the gait cycle. R-model: Redundant-Gait-Model. P-model: Plugin-Gait-Model. *p-value < 0.05, **p-value < 0.01

**Fig. 5:**
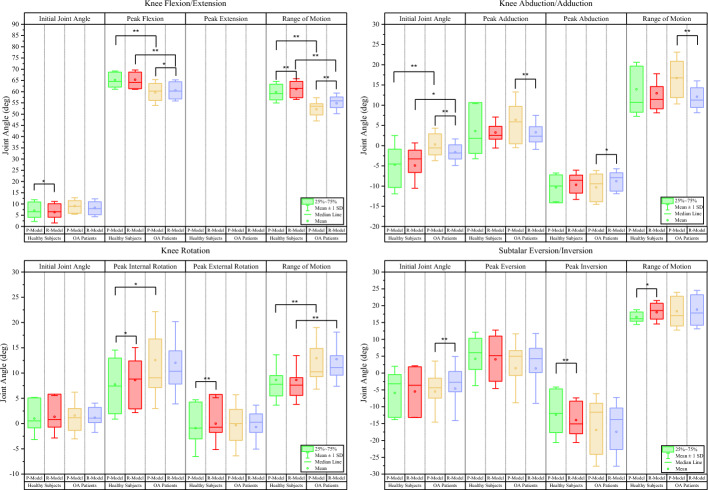
Box plot of the comparison of kinematic variables of the knee and subtalar joints across different marker sets and populations. Initial joint angle: joint angle when heel strike of the right leg. Range of motion: range of joint angle throughout the gait cycle. R-model: Redundant-Gait-Model, P-model: Plugin-Gait-Model. *p-value < 0.05, **p-value < 0.01

Figures 4 and 5 illustrate the kinematic variables between models and groups.

Kinematic variables that only showed significant differences between models in healthy subjects include peak hip internal rotation (*P* < 0.05), sagittal knee initial joint angle (*P* < 0.05), peak knee internal rotation (*P* < 0.05) and external rotation (*P* < 0.01), peak eversion (*P* < 0.01), and ROM (*P* < 0.05) of the subtalar joint.

In contrast, kinematic variables that only show significant differences between models in OA patients include sagittal hip initial joint angle (*P* < 0.01), peak hip flexion (*P* < 0.01) and extension (*P* < 0.01), peak knee flexion (*P* < 0.05), frontal knee initial joint angle (*P* < 0.01), and peak knee abduction (*P* < 0.05) and abduction (*P* < 0.01), frontal knee ROM (*P* < 0.01), and subtalar initial joint angle (*P* < 0.01).

Kinematic variables that showed significant differences between groups only for the R-Model was the ROM of the hip joint (*P* < 0.01) in the sagittal plane. In contrast, kinematic variables that showed significant differences between groups only for the P-Model was peak internal rotation (*P* < 0.05) of the knee joint.

### Intra-subject variation between models

In healthy subjects, R-model tended to show less variation in Hip Flexion/Extension, Hip Abduction/Adduction, and Knee Rotation (Table 2). In OA patients, R-model shows less variation in all the joint angles. However, no significant difference was found between marker sets in both healthy subjects and OA patients.

**Table 2:** Intra-subject MAV for angles at the hip, knee, and ankle joint in OA patients and Healthy Subjects

	OA Patients			Healthy Subjects		
	P-model	R-model	*P*-value	P-model	R-model	*P*-value
Hip Flexion/ Extension (°)	2.47 (0.69)	2.41 (0.68)	0.46	2.67 (1.46)	1.92 (0.55)	0.07
Hip Abduction/ Adduction (°)	2.63 (2.38)	1.83 (0.56)	0.32	1.29 (0.38)	1.26 (0.40)	0.54
Hip Rotation (°)	4.36 (3.32)	3.57 (1.62)	0.35	1.76 (0.47)	1.93 (0.60)	0.26
Knee Flexion/ Extension (°)	3.55 (1.54)	3.45 (1.27)	0.58	2.81 (0.51)	2.86 (0.48)	0.49
Knee Abduction/ Adduction (°)	3.76 (2.67)	2.87 (1.57)	0.16	2.12 (0.65)	2.14 (0.76)	0.92
Knee Rotation (°)	2.97 (4.90)	1.21 (0.49)	0.31	0.98 (0.45)	0.93 (0.46)	0.14
Ankle Plantarflexion/ Dorsiflexion (°)	2.99 (1.54)	2.86 (1.45)	0.34	1.94 (0.31)	1.92 (0.37)	0.70
Subtalar Eversion/ Inversion (°)	3.21 (1.56)	3.18 (1.54)	0.88	2.29 (0.75)	2.53 (1.05)	0.29

The values are mean (SD)

## Discussion

The aim of this study was to investigate the effect of different marker sets on joint kinematics using IK. When comparing the differences between models within each group, the joint angle at some points during the gait cycle demonstrates significant differences at the hip, knee, and ankle for both groups. Additionally, some of the kinematic variables also show significant differences between the two models. These findings support the first hypothesis. When comparing the differences between groups within each model, some kinematic variables only demonstrate significant differences between groups in R-model or P-model, which agrees with the second hypothesis.

Specifically, in the frontal and transverse plane, the differences between the two models were demonstrated for the ROM of Hip Rotation in both groups and Knee Abduction/Adduction in OA patients, the initial joint angle of the Knee Abduction/Adduction and Subtalar Eversion/Inversion in OA patients, Peak Internal Rotation of the Hip Rotation in healthy subjects, Peak Adduction and Peak Abduction of the Knee Abduction/Adduction in OA patients, Peak Internal Rotation and Peak External Rotation of the Knee Rotation in healthy subjects, Peak Inversion in healthy subjects. These findings are comparable with previous studies [19–21, 24, 29]. In previous studies, some researchers found the differences between marker sets at the knee and hip rotation in the transverse plane [19, 24, 25, 29], while others also found the differences at the knee in the frontal plane [24, 25, 29]. In this study, the findings in healthy subjects are in agreement with the former, while the findings in OA patients correspond to the latter. Therefore, different marker sets indeed affect the kinematic output in the frontal and transverse plane for gait analysis, and the degree of influence is related to the population.

In the sagittal plane, there are no differences between marker sets at the hip in healthy subjects, again the same as in previous studies [19, 24, 25, 29]. However, the Initial Joint Angle, Peak Extension and ROM at the knee, Peak Dorsiflexion, and ROM at the ankle show the differences between marker sets. In OA patients, more kinematic variables demonstrate the differences between marker sets. Namely, the Initial Joint Angle, Peak Flexion, and Peak Extension at the hip, Peak Flexion and sagittal ROM at the knee, and Peak Dorsiflexion and ROM at the ankle. These findings have not been reported to date. One reason may be that the variation of the mean of most kinematic variables from different marker sets do not exceed 5°. Thus, these differences did not attract enough attention (Fig. 2). Although the means are close, they indeed show significant differences when conducting statistical analysis. Therefore, the marker sets could also affect the kinematic variables at the hip, knee, and ankle in the sagittal plane.

Significant differences but close mean of kinematic output between marker sets may seem meaningless. However, when talking about our second hypothesis, it makes a difference. In this study, the difference of ROM at the hip in the sagittal plane between populations shows significance using the R-model while no significance using the P-model, indicating that the selection of the gait model could change the conclusion when comparing the kinematic output between populations. Similar findings also occur in the Peak Internal Rotation at the knee.

Since the selection of the gait model indeed affects the results when conducting gait analysis, the model selection is crucial. In this study, it could be found that the MAV of the R-model is smaller, which is in agreement with previous study [20]. Therefore, R-model has the potential to reduce variability. Moreover, marker misplacement often happens in gait analysis and could cause a deviation of 25° [9]. The selection of the R-model and IK might minimize the error to the greatest extent since IK and R-model are both robust to marker misplacement and motion artifact [14, 20, 30].

There are some limitations to this study. Firstly, the sample sizes are small, which might limit the comparison between models or groups. Secondly, the estimation of segments' pose is based on IK, which is global optimization. Therefore, the differences in kinematic variables between models or groups are not predictable. Thirdly, the R-model required lots of extra markers and increased the preparation time for the experiment.

## Conclusions

The findings in this study reveal that the selection of marker sets could affect not only the kinematic variables at the hip and knee in the non-sagittal plane but also the kinematic variables at the hip, knee, and ankle in the sagittal plane. Additionally, the selection of marker sets could also change the results when comparing the kinematic differences between different populations. Therefore, these findings demonstrate the need of increased caution on the selection of marker set when conducting gait analysis. Compared with the P-model, the R-model has the advantage of small variation and robustness to motion artifacts, marker misplacement, and even marker occlusion. Thus, the R-model may be a better choice for gait analysis and has the potential to be a popular gait model due to its strength over weakness.

## Data Availability

Not applicable.
